# In search of the optimum structural model for Internet Gaming Disorder

**DOI:** 10.1186/s12888-021-03148-8

**Published:** 2021-04-01

**Authors:** Vasileios Stavropoulos, Rapson Gomez, Mark D. Griffiths

**Affiliations:** 1 Institute for Health and Sport, Victoria University, Footscray, Melbourne, Australia; 2 Federation University, Ballarat, Australia; 3 Nottingham Trent University, Nottingham, UK

**Keywords:** Internet gaming disorder, Factor structure, Confirmatory factor analysis, Latent class analysis, Factor mixture modelling

## Abstract

**Background:**

Internet gaming Disorder (IGD) constitutes a recently proposed clinical disorder (American Psychiatric Association, Diagnostic and statistical manual of mental disorders, 2013). The present study examined if IGD is best conceptualized as categorical (present/absent), or dimensional (severity ranging from low to high), or both (i.e., hybrid of categorical/dimensional).

**Methods:**

Ratings of the nine DSM-5 IGD symptoms, as presented in the Internet Gaming Disorder Scale 9-Short Form (Pontes & Griffiths, Comput Hum Behav 45:137-143, 2015), from 738 gamers, aged 17 to 72 years, were collected. Confirmatory factor analysis (CFA), latent class analysis (LCA), and factor mixture modelling analysis (FMMA) procedures were applied to determine the optimum IGD model.

**Results:**

Although the findings showed most support for a FFMA model with two classes and one factor, there was also good statistical and substantive support for the one-factor CFA model, and the LCA model with three classes.

**Conclusion:**

It was concluded that while the optimum structure of IGD is most likely to be a hybrid model (i.e., concurrently categorical and dimensional), a uni-dimensional model and/or a three-class categorical model are also plausible.

**Supplementary Information:**

The online version contains supplementary material available at 10.1186/s12888-021-03148-8.

## In search of the optimum structural model for Internet Gaming Disorder

Disordered gaming has emerged as a contemporary psychopathological concern that has led to formal theoretical conceptualizations and diagnostic definitions [1]. In the 11th revision of the *International Classification of Diseases* [44], Gaming Disorder referring to clinically problematic gaming, occurring not necessarily online, was acknowledged as a formal diagnostic classification of a behavioral addiction. The latest (fifth) edition of the *Diagnostic and Statistical Manual of Mental Disorders* (DSM-5 [1]) included Internet Gaming Disorder (IGD) as a tentative disorder requiring further examination. To this end, a critical forward step is to validate the latent structure of DSM-5 IGD symptoms. This was the major goal of the present study.

## Internet Gaming Disorder in the DSM-5: conceptualization, assessment, correlates, and factor structure

### Conceptualization of IGD

The DSM-5 [1] defines IGD as an excessive activity involving the persistent and recurrent internet use to play videogames, resulting to considerable impairment or distress over a period of 1 year. It has nine symptoms involving preoccupation, withdrawal, tolerance, unsuccessful attempts to control usage, loss of interest in other activities, functional impairment, deception, escaping reality, and/or mood-relief in relation to Internet games’ usage [1]. Diagnosis requires at least five of these symptoms together with one’s significant impairment over a period of 12 months. IGD is diagnosed in terms of three specifiers, based on severity: mild, moderate, and severe presentations, However, it should be noted DSM-5 considers IGD as tentative, requiring more thorough empirical evidence to be fully recognized as a bona fide diagnosis [1].

### Assessment of IGD

To date, several psychometric scales based on the DSM-5 IGD criteria have been developed to facilitate the screening of IGD (e.g., [19, 22, 32]). One such measure (also used in the present study) is the nine-item Internet Gaming Disorder Scale – Short-Form (IGDS9-SF [32]). The IGDS9-SF [32] is arguably the most broadly utilized scale currently in the field of disordered gaming based on the number of studies using it and the number of languages it has been translated into [16, 45]. It can also be used with children and adults [45]. It is based on the symptoms and criteria for IGD in DSM-5 and assesses the severity of the nine IGD symptoms referring to the repercussions of online gaming usage over the past year. Each item of the IGDS9-SF matches an IGD DSM-5 criterion and is scored on a five-point Likert scale (from ‘never’ to ‘very often’).

In the initial scale development and validation study of the IGDS9-SF that involved a large and heterogeneous cohort of English-speaking adolescents and adult online gamers, Pontes and Griffiths [32] confirmed (using both exploratory factor analysis [EFA] and confirmatory factor analysis [CFA]) support for a single IGD factor. There was also evidence of very good validity based on the relationships with other variables (criterion-related evidence) and evidence in relation to its very good internal consistency reliability (Cronbach’s alpha ≥ .80). Many further CFA studies (e.g., [27, 33, 35, 37, 41, 45]) have confirmed the one-factor structure of the IGDS9-SF across different genders, countries, populations of gamers, and over time [10, 36, 37]. Regarding cut-off diagnostic thresholds for the IGDS9-SF, Pontes and Griffiths ([33]; see also [35]) advocated that meeting five or more of the nine the IGDS9-SF items, based on ‘often’ and ‘very often’ responses, provides a diagnostic indication in line with the DSM-5 proposals [1].

### Correlates of IGD

As frequent engagement with internet gaming is a defining feature of IGD, a positive association is expected with more time spent on internet gaming and IGD. Past studies have supported this association [25]. Studies have also examined the associations of age and gender with IGD. In relation to age, no significant associations have been reported by more recent studies (e.g., [28]). The findings for gender have been less consistent. Although most studies have reported higher prevalence of IGD among males [18, 21], there are studies that have reported no difference [3, 17], and also high involvement in excessive gaming among females [23]. Additionally, a robust finding is that IGD is highly comorbid with a range of psychopathologies. Out of 24 studies included in a meta-analysis (aimed at examining the association between IGD and psychopathology) reported by González-Bueso, Santamaría, Fernández, Merino, Montero, and Ribas [13], significant correlations between IGD and anxiety, depression, attention deficit hyperactivity disorder (ADHD), and obsessive-compulsive symptoms were reported in 92, 89, 85, and 75% of the studies, respectively, with the associations with anxiety, depression and ADHD being generally of high magnitude.

Although there are increasing data related to assessment and psychopathology correlates of IGD, more clarity is required regarding its latent structure. This is important, as a basic prerequisite for meaningful research involving any clinical disorder is the knowledge and understanding of its factor structure. Broadly, the factor structure of any psychopathological construct could be categorical (i.e., disorder present/disorder absent), or dimensional (i.e., ranging from low to high), or categorical/dimensional (i.e., a hybrid of the two). At the statistical level, CFA can be used to test dimensional models, latent class analysis (LCA; also called latent profile analysis, LPA) can be used to test categorical models, and factor mixture models can be used to test hybrid models. Details of CFA, LCA and FMMA are provided below.

### Factor structure of IGD

Most of the studies in this area have used CFA. CFA can test for dimensional structure. It employs the common-factor model to represent a number of regression calculations, where the observed/assessed items/criteria are regressed onto a latent dimension (in this case IGD). This enables the latent factor to be informed by the shared variances of the composing indicators/items/criteria. CFA studies have provided empirical support for a one-factor IGD model (e.g., [7, 26, 33, 35, 37, 45]).

LCA studies have also been used to examine the structure of IGD symptoms. LCA can test for categorical structure. LCA hypothesizes distinct categorical latent structures reflecting different profile memberships that underpin the covariance among the measured items. Individuals are classified into their most probable profile according to their responses’ variations. Consequently, individuals classified under the same profile are more the same than individuals classified under different profiles. Moreover, traditional LCA hypothesizes that assessed indicators-items within each profile do not covary, and that individuals of the same profile do not systematically vary considering the symptoms experienced (i.e., tend to be more homogenous). In sum, traditional LCA can identify profiles, but not variations within the profiles, as would a dimension enable. The number of profiles, suggested by the LCA, reflects the optimum number of the potential types (e.g., maximum data fit) of the disorder that best describe the population examined.

LCA findings have suggested the existence of various IGD typologies [4, 34]. More specifically, Pontes et al. [34] suggested five IGD typologies defined as “casual”, “regular”, “low risk”, “high risk” and “disordered gamers”. Similarly, a more recent study by Carras and Kardefelt-Winther [4] also contended on the existence of five IGD typologies/profiles (after applying LCA on 10 items corresponding to DSM-5 IGD symptoms and six dichotomous items assessing problems linked with videogame playing). These were different to Pontes et al. [34] and named as “normative” (featured by low endorsement probabilities of symptom/problem criteria), “IGD” (featured by high endorsement probabilities of symptom/problem criteria), “concerned” (featured by few or no symptoms whilst feeling that game-usage had precipitated/perpetuated difficulties in diverse life domains); “at risk” (featured by moderate endorsement probabilities of symptom criteria and high endorsement probabilities of problem criteria); and “engaged” (featured by moderate endorsement probabilities of symptom criteria and low endorsement probabilities of problem criteria [4]). Interestingly, the different typologies/classes suggested by Pontes et al. [34] and Carras and Kardefelt-Winther [4] differed from those in the DSM-5 IGD specifiers (i.e., mild, moderate, and severe [1]).

Taken together, the support for the one-factor model and the LCA models imply that the structure of IGD can be viewed in both dimensional and categorical terms. These findings also raise the possibility that IGD could be both dimensional and categorical at the same time. This can be assessed using Factor Mixture Modeling Analysis (FMMA). FMMA merges LCA and FA (or CFA) into one process (see the *‘**Statistical analyses**’* section for more detailed information). Therefore, it enables the representation of the underpinning latent construct in a hybrid way, both categorical and dimensional. However, compared to traditional LCA (which assumes zero correlations among the criteria/items), these may be correlated within the same class in FMMA. Such co-variations are represented utilizing factor analysis, and these (within-class factors/dimensions) may represent differences of the latent dimension among individuals classified under the same profile. Consequently, FMMA is a very useful technique in providing evidence-based validation of psychopathological constructs. In this context, there have several empirical FMMA findings referring to the latent structure of different psychological classifications (e.g., ADHD [12, 38];). However, to the best of the authors’ knowledge, no previous empirical research has investigated the latent structure of IGD using FMMA.

#### Aims of the present study

Based on the aforementioned literature, the goal of the present study was to establish, the optimum DSM-5 IGD structural model using LCA, CFA, and FMMA. Participants were a group of adults from the general Australian community, who were assessed online. The procedure illustrated by Clark et al. [5] in their tutorial paper on FMMA was used. The DSM-5 IGD symptom scores were obtained using ratings for the IGDS9-SF [32]. As mentioned above, daily hours spent on a weekday on preferred videogame, anxiety, depression, stress, inattention (ADHD-IA), and hyperactivity-impulsivity (ADHD-HI) have shown clear positive associations with IGD. Gender has shown mixed associations with IGD, and age has not shown significant association with IGD in more recent studies. Therefore, for the best CFA, LCA and FMMA models assessed, we examined (where appropriate) how the classes compared on their gender distributions, age, daily hours spent on preferred videogame, anxiety, depression, stress, ADHD-IA, and ADHD-HI. We also examined (where appropriate) how IGD factors in the models were associated with these variables.

In terms of predictions, and based on past findings, support was expected for the one-factor IGD model. We also expected support for categorical models. Based on a dichotomous view of the IGD symptoms (i.e., present/absent), at least two classes can be envisaged. Based the severity specifiers proposed for IGD in DSM-5 (mild, moderate, and severe), three classes can also be hypothesized. Additionally, it was assumed that the classes with higher endorsement of the IGD symptoms would be associated with higher levels of hours spent on preferred videogame, anxiety, depression, stress, ADHD-IA and ADHD-HI. Also, potential IGD factors in the models would be positively associated with these variables. We did not expect association for age with IGD. Finally, given mixed findings, we did not make any prediction for the association between gender and IGD.

## Method

### Participants

A convenience online community sample comprised 738 adult Australian online gamers, with ages varying from 17 to 72 years (mean = 25.22 years; *SD* = 7.67 years) was used for the present explorative secondary analysis study. The 95% confidence interval maximum sampling error (z = 1.96) for the current sample was estimated at + − 3.61%. The sample included 374 males (50.7%; mean age = 25.24 years, *SD* = 7.76 years), and 364 females (49.3%; mean age = 25.21 years, *SD* = 7.60 years). No significant age differences were reported across genders, (*t* [736] = *p* > .05).

Supplementary Table S1 presents supplementary information concerning the sample. In brief, over half of the respondents reported being employed, having attended technical or university education, and being currently in a romantic relationship. Additionally, respondents completed on average at least 12 school years, and spent about 5 years playing their preferred videogame, at an average of approximately 3.5 h per week. A quarter of participants reported that their partner also participated in their preferred videogame. With reference to the DASS-21 scores, the average presentations for depression (7.41 out of 21), anxiety (5.37 out of 21), and stress (6.86 out of 21) were less than one *SD* from the mean, around 1.5 *SD* from the mean, and just above one *SD* from the mean, respectively. Considering ADHD-related comorbidities, and with reference to Kessler et al. [15], a composite performance of 24 or more (out of 36) for ADHD-IA or ADHD-HI is indicative of an ADHD diagnosis. Participants in the present study had average total scores of 14.43 for ADHD-IA and 14.28 for ADHS-HI. For IGD, 66 participants (8.9%) exceeded the cut-off diagnostic threshold for the IGDS9-SF proposed by Pontes and Griffiths [33]. Therefore, the sample comprised mainly online gamers with non-pathological levels of psychopathology and gaming problems.

### Measures

#### Internet gaming disorder scale – short-form (IGDS9-SF **[**32**];**)

The IGDS9-SF was used to assess DSM-5 [1] IGD symptoms. The IGDS9-SF was originally introduced for assessing the nine DSM-5 symptoms with a time reference of the past year. An example item is: “Have you lost interests in previous hobbies and other entertainment activities as a result of your engagement with the game?” Items are responded to on a five-point scale from 0 (‘Never’) to 4 (‘Very often’). Item scores are summed resulting in a final score between 0 and 36 with higher final scores indicating higher IGD severity. Overall, given its good psychometric properties, its alignment with the symptoms for IGD in DSM-5, and its wide acceptance, the IGDS9-SF is a useful psychometric instrument for research and clinical use in IGD research [32], including the exploration of its optimum factor structure utilizing FMMA processes. In the present study, the internal reliability for the IGDS9-SF instrument was very good (Cronbach *α* = .84).

#### Adult ADHD self-report scale symptom checklist (ASRS [15];)

The ASRS comprises the 18 DSM-5 ADHD symptoms: nine assessing ADHD-IA symptoms and nine assessing ADHD-HI symptoms. An example ADHD-IA item is: “How often do you make careless mistakes when you have to work on a boring or difficult project?” An example ADHD-HI symptom is: “How often do you fidget or squirm with your hands or feet when you have to sit down for a long time?” For all items, participants indicate how often they have experienced each symptom over the past 6 months on a scale with options of 0 (“never”), 1 (“rarely”), 2 (“sometimes”), 3 (“often”), and 4 (“very often”). Item scores are added per dimension resulting in a scoring range between 0 and 36, with lower scores indicating lower symptom levels. In the present study, the internal reliabilities for the ADHD-IA and ADHD-HI dimensions were good to very good (*α* = .85 and *α* = .77 respectively).

#### Depression anxiety stress scales-21 (DASS-21 [24];)

Depression, anxiety and stress were assessed using the 21-item DASS-21. The DASS-21 comprises three seven-question sub-scales for depression, anxiety, and stress [24]. An example of a depression item is: “I couldn’t seem to experience any positive feeling at all”. An example of an anxiety item is: “I experienced breathing difficulty”. An example of a stress item is: “I found it hard to wind down”. For all items, participants are required to indicate how often they experienced them in the past week, utilizing a four-point Likert scale from 0 (“did not apply to me at all”) to 3 (“applied to me very much or most of the time”). Scores of the items relating to each subscale are added resulting to a final score of 0–21 per scale with higher scores indicating higher symptom levels. Past evidence has advocated sufficient convergent and discriminant validity, and high internal reliability consistencies across the DASS-21 subscales [24, 30] (Lovibond & Lovibond, 95, [31]). In the present study, the depression, anxiety, and stress scales’ internal reliabilities were good to very good (*α =* .84, *α =* .71 and *α =* .83, respectively).

### Procedure

The data collection used in the present explorative secondary analysis study was approved by the research team’s university Human Research Ethics Committee, prior to commencing in December 2018. The survey investigated the associations between IGD behaviors, ADHD, depression, anxiety and stress. Given that the study targeted gamers’ experiences, self-report measures were utilized, and online gaming communities were targeted. The a priori analyses involving F tests (linear multiple regression: fixed model, R^2^ deviation from zero), with an effect size (f^2^) of .15, an alpha (α) error probability of .05, and power of .95, indicated that a minimum sample size of 138 participants were required. The DASS-21 and the ASRS responses included in the data have been previously used by Gomez, Stavropoulos and Griffiths [11] to address the factor structure of the DASS-21 via confirmatory factor analysis and exploratory structural equation modelling. This previous data analysis did not overlap with the present study’s aims and did not include any data relating to the IGDS9-SF scores. Prospective respondents had to be internet gamers over 18 years of age. The online survey link was distributed across media platforms (e.g., *Facebook*), online interactive forums (e.g., *Discord*) and online gaming communities, as well as using advertisement flyers. The independent distribution of the survey link among gamers was additionally encouraged. Data collection was arranged such that participants could not skip items to prevent missing values. Thus, only completed surveys were included in the present analyses. The *SurveyGizmo* application was used to collect the data. Interested gamers initially had access to the Plain Language Information Statement (PLIS). This clarified the voluntary and anonymous participation and requested the online provision of written (digitally recorded) informed consent. No penalties applied for withdrawal. Online collection was selected because it is an effective way to engage hard-to-reach populations and especially online gamers).

### Statistical analyses

All statistical analyses were conducted using M*plus* Version 7 [29]. As aforementioned, the models and procedures used in the present study aligned very closely to that in the tutorial FMMA paper by Clark et al., [5]. This approach entails first fitting LCA, FA, and FMMA to the data. Robust maximum likelihood (MLR) was employed for all CFA, LCA and FMMA calculations. As noted by others (e.g., [39]), when there are more than four response categories, MLR can be confidently applied, and there is no need to use an estimator specifically for ordered-categorical scores. Supplementary Table S2 provides the frequencies of endorsements of the five different response categories for the nine items of the IGDS9-SF. To secure convergence on the optimal LCA and FMMA models, 500 random sets of starting values and 100 iterations were initially enabled, and 20 optimizations-improvements were additionally applied.

### Modeling and evaluating the fit of the CFA model

Given robust support for the one-factor IGDS9-SF model, we used CFA to test support for a one-factor IGD model. Consequently, we tested a CFA model with all nine IGD items loading on a single factor. Because chi-square values are inflated by large sample sizes, we also used approximate indices. These were the root mean squared error of approximation (RMSEA), the comparative fit index (CFI), and the Tucker Lewis Index (TLI). Hu and Bentler [14] recommend that RMSEA values close to 0.06, or lower, should be taken as good fit, 0.07 to 0.08 as moderate fit, 0.08 to .10 as marginal fit, and > .10 as poor fit. Considering CFI and TLI scores, those closer to .95 and higher indicate good fit, and those closer to .90 and .95 are deemed sufficient.

### Building LCA and FMMA models

In the LCA, all nine ICD items were included uncorrelated within the different profiles. For this analysis, the number of classes is increased sequentially by one in each instance. Once the LCA and CFA models are computed and the best models are selected, FMMA models are fitted. This starts with the two classes and one factor. For building the FMMA models, the number of factors is increased sequentially by one in each instance. Once these models are computed, the computation reverts back to the three classes and one factor. The number of factors is again increased sequentially by one in each instance. This process is repeated in sequence with more classes, increasing by one class each time. The number of factors is increased by the number of factors in the optimum FA model, and the number of classes is increased by the number of classes in the optimum LCA model. The FMMA model building steps are applied separately for all four variants of FMMA (FMMA-1, FMMA-2, FMMA-3, and FMMA-4) models. These variants are described in supplementary Table S3. Finally, having established the best FA, LCA and FMMA models, the best overall model is established. For this, the best FMMA model is compared with the best FA and LCA models.

### Selecting the best LCA and FMMA models

At present, there is no commonly accepted method for selecting the best number of classes in LCA and FMMA models. In this paper we used the approach recommended and illustrated by Clark et al. [5] that is in turn based on others (e.g., [31]). This approach recommends using both statistical and substantive model checking. For statistical comparison of models, the Lo-Mendell-Rubin Test (LMRT), and the Bayesian Information Criterion (BIC) are used. LMRT compares the model fit of a model of specific number of profiles with that of a model of one profile less. A non-significant *p*-value suggests lack of improvement with the addition of one class. For BIC, lower values indicate better fit. Selection of the best LCA and FMMA model proceeds as follows. Starting with the model with the lowest BIC, the *p*-value for the LMRT is checked. If it is significant then it means that a model with one class model less is a better model. Therefore, the *p*-value for the LMRT of the model with the next lowest BIC is checked. If this is significant, the next lowest BIC is checked for the *p*-value for its LMRT value. This process is continued until there is a model in which the *p*-value for the LMRT of the model is not significant. This could indicate the best LCA or FMMA model. It is important to note that LMR can only be used to compare models that have the same parameterization, but differing numbers of classes.

### Selecting the best overall model

The LMRT cannot be used to compare different model types (FA, LCA, and FMMA). Therefore, the comparison of the best FA, LCA and FMMA models is based on their BIC values, with the model with the lowest BIC values adopted as the best overall model. By this point, this model could have already been established as substantive and interpretable.

### Examining the substantive values of the classes and factors in the best fitting models

The final acceptance of CFA, LCA and FMMA models as best models will depend on them also showing substantive interpretation. Generally, this involves examining if the factors and/or classes in the model have substantive meaning. This should involve examining if the factors and classes are theoretically meaningful, such as theoretically expected relations with external variables. With M*plus*, for LCA and FMMA models, it is possible to obtain, for each individual, the most probable class to which the individual belongs. Using this information for class membership, we can examine if the individuals in the different classes in the best fitting LCA and FMMA model differ for gender distribution, age, hours spent on their preferred videogame, anxiety, depression, stress, ADHD-IA, and ADHD-HI. As noted earlier, there are now ample data showing that IGD (i) is associated positively with hours spent on their preferred videogame, anxiety, depression, stress, ADHD-IA, and ADHD-HI, (ii) has no association with age, and (iii) mixed findings with regard to gender. For each of these variables (except gender), the plan was to compare for class differences using either one-way ANOVAs (if three or more classes were present) or t-test (if only two classes were present). For gender, its distributions across the classes were examined using the chi-square test. Additionally, we also examined the associations of gender, age, hours spent on preferred videogame, anxiety, depression, stress, ADHD-IA, and ADHD-HI with the factors in the best fitting CFA and FMMA models. This was done by regressing these variables on the IGD factors in the models.

## Results

### Fit for the one-factor model CFA

The fit values from the CFA for the one-factor model for the IGDS9-SF were as follows: MLR*χ*^2^ (*df* = 27) = 138.08, *p* < .001; RMSEA = .075 (90% CI = .063–.087); CFI = .951, and TLI = 0.945. Based on Hu and Bentler’s [14] guidelines, the CFI and TLI values can be interpreted as showing good fit, and RMSEA value as showing moderate fit for the unidimensional IGD structure. Additionally, all symptoms in the model loaded significantly (*p* < .001) and saliently (ranging from .483 to .920 on the IGD factor). Table 1 shows the standardized path coefficients for the predictions of the external variables (i. e. gender distribution, age, hours spent on preferred videogame, anxiety, depression, stress, ADHD-IA, and ADHD-HI) by the IGD factor. As shown, except for age and gender, the standardized path coefficients for all other variables were significant and positive. These associations were as theoretically expected. Therefore, the one-factor CFA model for IGD had substantive meaning.

**Table 1:** Standardized coefficients of the predictors of the background and psychopathology variables by the IGD factor in the CFA and FMMA models

Predictors	CFA	FMMA	
		Class 1	Class 2
Gender	−0.054	.040	−0.087
Age	−0.063	−.039	−0.162*
Hours/week on preferred games	0.250***	0.191***	0.352***
Inattention	0.490***	0.429***	0.466***
Hyperactivity/impulsivity	0.460***	0.408***	0.520***
Stress	0.441***	0.355***	0.538***
Anxiety	0.384***	0.301***	0.456***
Depression	0.449***	0.360***	0.509***

***p < .001, **p < .01, *p < .05

### LCA models

Table 2 summarizes the BIC, LMRT, the bootstrapped LRT indexes for the LCA and the FMMA models assessed in the present study. Considering the LCAs, one-to-four profile models were evaluated. The four-class LCA model had the lowest BIC values. However, its *p*-value for LMRT was significant, thereby suggesting that a class with one less model (three-class LCA model) would be preferable. The model with the second lowest BIC value was the three-class LCA model, and the *p*-value for the LMRT for this model was not significant. Given these findings, the three-class LCA model was tentatively considered as the best LCA model. To evaluate further the substantive value of this model, the IGD symptom profiles of the classes in this model were examined. The findings indicated a class with high endorsement on most of the IGD symptoms, a class with low endorsement on most of the IGD symptoms, and an intermediate class with symptom profiles between the high and the low endorsement classes. As noted earlier, DSM-5 suggests IGD should be specified in terms of mild, moderate, and severe presentation types according to the degree of the IGD symptoms present [1]. These types can be reinterpreted in the present study as low, intermediate, and high classes, respectively.

**Table 2:** CFA, LCA and FMMA: model comparisons and fit indices

No. of classes (c)/factors (f)	LL	No. of parameters	BIC	*p* for LMR	Entropy
Confirmatory factor analysis					
One-factor	− 8789	27	17,757		
Latent class analysis					
One-class	−10,067	18	20,254		
Two-class	− 9175	28	18,536	0.0000	0.903
Three-class	− 8795	38	17,842	0.0482	0.953
Four-class	− 8242	48	16,801	0. 2489	1.000
Factor mixture model					
Two-class/one-factor					
FMMA-1	−9175	28	18,536	0.0000	0.803
FMMA-2	− 8750	30	17,698	0.0104	0.636
FMMA-3	− 8533	38	17,317	0.0000	0.984
FMMA-4	− 8509	48	17,336	0.0000	0.977
Three-class/one-factor					
FMMA-1	− 8809	31	17,824	0.0000	0.951
FMMA-2	− 8719	33	17,657	0. 1422	0.983
FMMA-3	− 8481	49	17,286	0. 2349	0.964
FMMA-4	− 8400	68	17,249	0. 1223	0.948

*LL* Log Likelihood, *BIC* Bayesian Information Criterion, *LMR* Lo–Mendell–Rubin

There were 226 (60.4%), 126 (23.4%) and 23 (6.1%) males in the low, intermediate, and high endorsement classes, respectively; and there were 219 (60.2%), 110 (30.2%) and 35 (9.6%) females in the low, intermediate, and high endorsement classes, respectively. The results of the chi-square test indicated no significant difference[χ^2^ (df = 2) =3.41, *p* = 0.181] regarding the distribution of the two genders across the classes. Table 3 shows the results of the comparisons across the three classes for age, hours spent each week on preferred videogame, anxiety, depression, stress, ADHD-IA, and ADHD-HI. As shown, except for age, the high endorsement class scored significantly higher than the intermediate endorsement, which in turn scored higher than the low endorsement class. The differences were as theoretically expected.

**Table 3:** Comparisons of the mean of the low (*N* = 626) and high (*N* = 112) classes for all continuous predictors

Predictors	Low (L)	Intermediate (I)	High (H)	*t* (*df*)	Δ Class	Eta squared
Age	25.43 (7.78)	25.11 (7.76)	23.78 (6.15)	1.20	H = I = L	.003
Hours/week on preferred games	3.25 (3.10)	3.99 (3.25)	5.62 (4.12)	15.41***	H > I > L	.040
Inattention	12.82 (5.99)	16.26 (5.21)	12.62 (5.89)	72.78***	H > I > L	.165
Hyperactivity/ impulsivity	12.58 (5.56)	16.04 (5.56)	20.24 (6.40)	64.41***	H > I > L	.149
Stress	5.71 (3.93)	7.89 (4.11)	12.22 (4.76)	77.26***	H > I > L	.174
Anxiety	4.41 (3.80)	6.179 (4.31)	9.40 (4.85)	45.52***	H > I > L	.110
Depression	5.83 (5.07)	8.92 (5.44)	13.40 (5.03)	69.03***	H > I > L	.158

***p < .001, **p < .01, *p < .05. The *df* value for all *t* values = 736

Overall, the findings showed the three-class LCA model had substantive meaning. Consequently, this model was considered the optimum LCA model. For the model, the entropy (an index of membership classification clarity-accuracy) was high (.953), as were the posterior probabilities for the accurate classification across the three classes (.964, .979, and .986 for the high, intermediate, and low classes, respectively). The proportions of individuals in the high, intermediate, and low endorsement profiles were 7.9, 31.8, and 60.3%, respectively.

### FMMA models

Following the results of the CFA and LCA, for all FMMA models, one-to-three class solutions for the unidimensional IGD structure were examined. Therefore, for the two-class/one-factor, and three-class/one-factor model, the four versions of FMMA were assessed (i.e., FMMA-1, FMMA-2, FMMA-3, and FMMA-4). The one-class model would be equal to the one-factor structure.

Table 2 presents the results of all the FMMA models. As shown, the three-class/one factor FMMA-4 model had the lowest BIC value. However, the *p*-value for its LMRT value was not significant, thereby suggesting that a model with one class less could be a better model. The model with the second lowest BIC value was the three-class/one factor FMMA-3 model. The *p*-value for the LMRT value for this model was also not significant, thereby suggesting that this was not a suitable model. The model with the next lowest BIC value was the two-class/one-factor FMMA-3 model. The *p*-value for the LMR T_value_ of this model was significant. Therefore, from a statistical viewpoint, the two-class/one-factor FMMA-3 model was considered as the best fitting FMMA model. To evaluate further the substantive value of this model, the IGD symptom profiles of the two classes in this model were examined.

Table 4 shows the mean scores (and standard errors) and intercepts scores (and standard errors) for all IGD-9 symptoms in the two classes in the FMMA-3 variant of the two-class/one-factor model. It also includes the unstandardized factor loadings for the symptoms in this model. Because these loadings were invariant across classes, these loadings were applicable to both classes. Supplementary Figure S1 depicts the mean values (with their 95% confidence intervals) for each symptom in the two classes of this model. As can be seen in Table 3 and Figure S1, all nine symptoms were higher in one class (Class A) than the other class (Class B). For classes B and A, the mean scores of the symptoms ranged from 1.20 to 3.33, and 2.52 to 3.80, respectively. The overall mean (SD) for the B and A classes were 2.17 (0.65) and 3.28 (0.42), respectively. The intercepts values for the symptoms for the B and A classes ranged from 1.57 to 3.08, and 2.32 to 7.05, respectively. The overall mean (SD) for the B and A classes were 2.25 (0.525) and 3.28 (0.42), respectively. Therefore, the two classes in the FMMA-3 variant of the two-class/one-factor model can be viewed in terms of high (Class A) and low (Class B) levels of endorsements of IGD symptoms.

**Table 4:** IGDS9-SF item mean and intercept values for the FMMA3-Class one-factor model and factor loadings for all classes

	Low endorsement class				High endorsement class				All classes	
	Mean		Intercept		Mean		Intercept		Factor loadings	
Brief item description	X	*SE*	β	*SE*	X	*SE*	β	*SE*	ƛ	*SE*
Preoccupation (1)	2.89	0.05	2.66	0.07	3.77	0.09	3.09	0.13	1.00	0.00
Negative emotions (2)	2.11	0.05	1.85	0.04	2.96	0.13	2.22	0.11	1.26	0.09
Increasing time (3)	2.34	0.05	2.10	0.05	3.26	0.12	2.47	0.12	1.28	0.08
Lacking control (4)	1.91	0.04	1.95	0.04	2.99	0.12	2.65	0.14	1.02	0.07
Giving up activities (5)	2.23	0.03	3.08	0.09	3.21	0.09	3.39	0.18	1.12	0.07
Continuation (6)	2.00	0.04	1.80	0.04	3.39	0.12	2.70	0.14	1.08	0.08
Deception (7)	1.52	0.04	1.57	0.03	2.52	0.13	2.36	0.13	0.84	0.08
Escape (8)	3.33	0.05	2.76	0.08	3.80	0.11	2.88	0.13	0.96	0.09
Jeopardizing (9)	1.20	0.02	2.51	0.06	3.62	0.07	7.05	0.26	0.32	0.04

X = mean; β = intercept, SE = standard error

Table 5 provides the findings of the comparisons, using *t*-tests, across the two classes for age, hours spent on one’s preferred videogame, anxiety, depression, stress, ADHD-IA, and ADHD-HI. Prior to this, chi-square was used to examined difference gender distribution across the classes. There were 292 (46.6%) and 72 (64.2%) females in the low and high endorsement classes, respectively; and there were 334 (53.4%) and 40 (35.7%) males in the low and high endorsement classes, respectively. The results of the chi-square test indicated significant difference, (χ^2^ = 11.83, *p* < .01), with more females in the high endorsement class. The eta squared effect size for these differences was medium (η2 = .1) based on benchmarks for eta squared: small = 0.01, medium = 0.06, and large = 0.14 [6].

**Table 5:** Comparisons of the mean of the low (*N* = 626) and high (*N* = 112) classes for all continuous predictors

Predictors	Low (L)	High (H)	*t* (*df*)	Δ Class	Cohen’s *d*
Age	25.26 (7.93)	24.90 (6.45)	0.45	L = H	0.46
Hours/week on preferred games	3.51 (3.15)	4.57 (3.95)	3.16**	H > L	0.32
Inattention	13.76 (6.01)	18.16 (5.91)	7,15***	H > L	0.73
Hyperactivity/impulsivity	13.78 (5.88)	17.08 (6.50)	5.34***	H > L	0.55
Stress	6.45 (4.14)	9.13 (4.76)	6.17***	H > L	0.63
Anxiety	4.98 4.08)	7.49 (4.83)	5.82***	H > L	0.60
Depression	6.83 (5.45)	10.68 (5.66)	6.85***	H > L	0.70

***p < .001, **p < .01, *p < .05. The *df* value for all *t* values = 736

Table 1 also includes the standardized path coefficients for the predictions of gender distribution, age, hours spent on preferred videogame, anxiety, depression, stress, ADHD-IA, and ADHD-HI, by the IGD factors in Classes 1 and 2. As shown, except for age (in Class 1) and gender, the standardized path coefficients for all other variables were significant and positive. The associations for both classes were as theoretically expected. Therefore, the CFA IGD factor in both classes had substantive meaning. Overall, the two-class/one factor FMMA-3 model had substantive meaning. Consequently, this model was considered the optimum FMMA model. Its entropy was high (0.984), while the posterior probabilities of individuals correctly clustered in high- and low-severity profiles were 0.99, and 0.999, respectively. The number of participants classified in the high- and low-severity profiles were 112 (15.18%) and 626 (84.828%), respectively.

For this model, the mean ratings for six of the nine IGD symptoms for the class with high levels of endorsements were above 3. The three symptoms that did not have as high level of endorsement were negative emotions (symptom 2), lacking control (symptom 4), and deception (symptom 7). The mean ratings for these symptoms were 2.96, 2.99, and 2.36, respectively. However, these mean scores indicate that the ratings for negative emotions and losing-lacking control were both very close to 3. With the exception of one symptom (symptom 8 relating to escape, which had a rating mean value of 3.33), the mean ratings for all the other eight symptoms in the low endorsement class were below 3. Therefore, overall (and based on a symptom cut-off score of > 3 for inferring the presence of a symptom [33, 35];), the classes with high and low levels of endorsements can be viewed as classes that are affected and unaffected by IGD symptoms.

### Comparing the best CFA, LCA and FMMA model – selecting the overall best model

Across the three different types of models that were selected as the best models, the two-class/one-factor FMMA-3 model had had the lowest BIC value. Thereby, this was considered the best statistically fitting model for the IGDS9-SF.

## Discussion

The CFA analyses suggested statistically good and substantively meaningful support for the one-factor IGD structure. This indicates that the underlying structure of IGD can be conceptualized as a single dimension that is continuous and normally distributed. This result aligns with those of previous studies (e.g., [9, 27, 32, 33, 35, 37, 45]). The LCA findings indicated statistically good and substantively meaningful support for a three-class model, with classes for high (7.9%), intermediate (31.8%), and low (60.3%) endorsement. This model raises the possibility that IGD is categorical, with three categories, corresponding to low, intermediate, and high severities. Indeed, the classes in the three-class LCA model can be seen as corresponding with the three intensity specifiers suggested in DSM-5 [1]. These classes are consistent with existing LCA data involving items/symptoms examining internet addiction and smartphone addiction [20], as well as symptoms assessing IGD together with psychosocial wellbeing items [4]. The FMMA findings provided statistically good and substantively meaningful support for the FMMA-3 version of a uni-dimensional structure with two profiles. The support for this model suggests that the underlying structure of IGD is continuous, whilst not uniformly distributed the same way. Instead, there are two distributions, corresponding to the two classes. This means that the underlying structure of IGD can be simultaneously continuous and categorical. The two classes were low or unaffected (84.82%,) and the high or affected (15.18%) endorsement classes. Expressed differently, individuals with IGD can be separated into a high endorsement (affected) or low endorsement (unaffected) groups. Additionally, within each group individuals will have varying levels of severity. As pointed out in supplementary Table S3, in this model, the dimension mean-average scores are allowed to vary across the profiles (i. e. item loadings and thresholds were constraint equal between the profiles, and the within-profile co-variances were freely calculated). Therefore, the support for variant 3 FMMA model indicates that while members of two IGD classes use the same metric to response to the IGD items, they have different interpretations of the items. Moreover, while there is some heterogeneity between the classes considering the IGD presentations experienced, members of different classes differ primarily regarding their item endorsement probabilities (and not their average experience of IGD behavior). The overall classification accuracy (i.e., entropy; correct-clear membership of the participants across the classes) for this model was very high, showing that there are minimum overlaps between the two profiles suggested. This is further reinforced by the posterior probabilities of participants correctly allocated in each of the two profiles being additionally high. It should be noted that this is the first study that has applied the FMMA approach in examining the structure of IGD symptoms.

Across the three different types of models that were considered as the best models, the two-class/one factor FMMA-3 model had the lowest BIC value, thereby indicating that overall, this was the best fitting model for the IGDS-9-SF. However, the best fitting CFA model (one-factor model) and the best fitting LCA model (LCA model with three classes) were also substantively meaningful. Therefore, it is prudent to conclude that while the FMMA-3 two-class/one-factor model appears to be statistically the best of the models tested here, the CFA one-factor model and the LCA three-class model are also acceptable and meaningful models.

### IGD correlates

The findings in the present study showed strong associations for IGD with hours spent on preferred videogame, anxiety, depression, stress, ADHD-IA, and ADHD-HI. These findings were expected and are consistent with existing data (e.g., [13, 25]). They indicate that IGD is comorbid with a range of psychopathologies. Specifically, we found that IGD was associated with longer weekly engagement with playing of preferred online videogame. This finding is consistent with literature suggesting higher online absorption among individuals with IGD [25]. This higher level of absorption has been explained by past studies as being a result of: (i) the deeper engagement in the gaming activity, due to the progressive in-game leveling-up process that matches increasing gamer skills with increasing game challenges, generating a sense of online flow [2]; and (ii) the absorption by the animated online context, that is eventually misperceived as real, generating a sense of tele-presence [2].

Similar to some existing data [28], the one-factor CFA and LCA three-class models showed no association with age. For the FMMA-3 two-class/one factor model, age was also not associated with the IGD factor in Class 1. For Class 2, the IGD factor was associated negatively with age. Therefore, the findings raise the possibility that among individuals with high IGD symptoms, IGD is likely to be more severe among younger individuals. The one-factor CFA and LCA three-class models showed no association with gender. For the FMMA-3 two-class/one factor model, gender was also not associated with the IGD factor in Classes 1 and 2. However, there were more females than males in the high endorsement class. Although the findings for gender were difficult to interpret, it could be assumed that, as in the case of other disorders (e. g. ADHD), although fewer females tend to be involved with gaming, when they do, they tend to be more severely impacted [43]. On that basis, it is conceivable that although gaming might function as a more socially acceptable activity for males, it may be more of an addictive, dysfunctional emotional regulation strategy for females. This corresponds with recent literature highlighting the IGD vulnerability of females [23].

## Conclusions

The results and conclusions made in the present study should be evaluated in the light of certain limitations. First, the study utilized ratings of the IGD criteria collected utilizing the IGDS9-SF [32]. Therefore, it is questionable if varying outcomes would have occurred by utilizing different IGD psychometric scales or semi-structured interview collected information. Moreover, because all the gamers enrolled in our convenience sample were from a non-random community, the findings may be biased. Additionally, as they were all residents of a Western country, and were reasonably well-adjusted, it could be inferred that the results may not be generalizable to clinic-referred populations, IGD diagnosed samples, or to other cultural/national groups. Also, because we had no information on participants who knew of the study, but did not participate, we are not sure how this may have confounded the findings. Nevertheless, because a community sample was used, the analyses were able to model the full IGD trait spectrum. Finally, a construct (such as IGD) can be either reflective or formative [8]. For a reflective construct, the construct is seen as causing variations in the behavior indicators, whereas in a formative construct, this is reversed, with the behavior indicators seen as causing variation in the construct. The CFA, LCA, and FMMA models tested in the present study assumed that IGD is a reflective construct. However, this may not necessarily be the case. Indeed, van Rooij, Van Looy and Billieux [42] have supported that IGD can be conceptualized as a formative construct, rather than a reflective construct. This is an important distinction when validating a construct. As noted by van Rooij et al., [42] when a formative construct is viewed as reflective, the estimated parameters in the model could be biased. As we did not consider IGD to be a formative construct, it is possible that our findings and the interpretation made may be seen by some as biased, or even misleading. We therefore recommend that future studies examine the formative nature of the IGD construct. Roberts and Thatcher [40] have provided a useful tutorial on how to test such a question. Keeping in mind these limitations one might wish to consider the current findings as preliminary. However, the results from the present research advocate for more FMMA based research in this area, controlling for the limitations highlighted.

## Supplementary Information

**Additional file 1: Supplementary Table S1.** Frequencies and descriptive statistics of background variables collected in the study for all participants together. **Supplementary Table S2.** Frequencies of Endorsements of the Different Response Categories for the Nine Items of the IGDS9-SF. **Supplementary Table S3.** Description of the Different Variants of FMMA Models.

**Additional file 2.**

## Data Availability

The datasets used and/or analyzed during the present study is available in the current submission.

## Abbreviations

APA: American Psychiatric Association
DSM-5: Diagnostic and statistical manual of mental disorder 5th edition
ADHD: Attention deficit and hyperactivity disorder
IGD: Internet gaming disorder
IGDS9-SF: Internet gaming disorder scale 9-short form
LCA: Latent class analysis
FMMA: Factor mixture modeling
