# Supplementary material for: In search of the optimum structural model for Internet Gaming Disorder
Source: BMC Psychiatry. 2021 Apr 1;21:176. doi: 10.1186/s12888-021-03148-8 (PMC8015185; doi:10.1186/s12888-021-03148-8)
Supplement: Supplementary file 1 — Additional file 1: Supplementary Table S1. Frequencies and descriptive statistics of background variables collected in the study for all participants together. Supplementary Table S2. Frequencies of Endorsements of the Different Response Categories for the Nine Items of the IGDS9-SF. Supplementary Table S3. Description of the Different Variants of FMMA Models. [file 12888_2021_3148_MOESM1_ESM.docx]

**Supplementary Table S1**

*Frequencies and descriptive statistics of background variables collected in the study for all participants together*

| Variables | Frequencies/descriptive statistics |
| --- | --- |
| Employed | Frequency (percentage) = 629 (71.7%) |
| *Highest educational level* |  |
| Primary | Frequency (percentage) = 42 (5.7%) |
| Secondary | Frequency (percentage) = 268 (36.3%) |
| Technical | Frequency (percentage) = 193 (26.2%) |
| University | Frequency (percentage) = 235 (31.8%) |
| Is involved in romantic relationships | Frequency (percentage) = 405 (54.9%) |
| Participation of partner | Frequency (percentage) = 190 (25.7%) |
| Years of education | Mean (*SD*) = 12.01 (5.12); Ma/Min = 0/25 |
| Years playing preferred game | Mean (*SD*) = 4.88 (4.20); Ma/Min = 0/29 |
| Hours/week on preferred games | Mean (*SD*) = 3.67 (3.31); Ma/Min = 0/20 |
| ASRS - Inattention ( | Mean (SD) = 14.43 (6.20); Ma/Min = 0/32 |
| ASRS - Hyperactivity/impulsivity | Mean (SD) = 14.28 (6.09); Ma/Min = 0/36 |
| DASS - Stress | Mean (*SD*) = 6.86 (4.34); Ma/Min = 0/21 |
| DASS - Anxiety | Mean (*SD*) = 5.36 (4.29); Ma/Min = 0/21 |
| DASS - Depression | Mean (*SD*) = 7.41 (5.65); Ma/Min = 0/21 |

*Note*. ASRS = Adult ADHD Self-Report Scale Symptom Checklist; DASS = Depression Anxiety Stress Scales-21; *SD* = Standard Deviation; Max/Min = Maximum/Minimum.

**Supplementary Table S2**

*Frequencies of Endorsements of the Different Response Categories for the Nine Items of the IGDS9-SF*

| Item number | 0 = Never | 1 = Rarely | 2 = Sometimes | 3 = Often | 4 = Very Often |
| --- | --- | --- | --- | --- | --- |
| 1 | 72 | 175 | 242 | 159 | 90 |
| 2 | 261 | 208 | 151 | 71 | 47 |
| 3 | 185 | 208 | 204 | 87 | 54 |
| 4 | 269 | 255 | 129 | 61 | 24 |
| 5 | 83 | 369 | 222 | 51 | 13 |
| 6 | 284 | 183 | 152 | 67 | 92 |
| 7 | 465 | 125 | 93 | 33 | 22 |
| 8 | 67 | 102 | 210 | 187 | 172 |
| 9 | 498 | 128 | 64 | 27 | 21 |

**Supplementary Table S3**

*Description of the Different Variants of FMMA Models.*

FMMA-1

In the FMMA-1 (monoculture), the dimension mean-average scores were exclusively allowed to vary across the profiles (item loadings and thresholds were constraint equal between the profiles, while within-profile co-variances were fixed to 0), thereby suggesting that IGD is assessed in the same way-metric and using the same interpretation between the profiles (invariant structure), while the IGD profiles resulting are homogenous. Accordingly, members or one profile vary from those of another only considering their average IGD presentations. This additionally suggests that the potentially different IGD profiles equal to the number of classes revealed. Therefore, confirmation of this structure would be favorable of a more qualitative/categorical IGD conceptualization.

FMMA-2

The FMMA-2 is identical to the FMMA-1, with the difference of the within-profile factor covariance being freely calculated (and not restraint to 0). Confirmation of this model would suggest IGD being assessed on the same metric and maintaining the same interpretation between the different profiles. Further, it would propose that while members of different IGD profiles are different considering their average IGD presentations, there is additionally heterogeneity considering the IGD trait within the different profiles (Clark et al., 2013).

FMMA-3

The FMMA-3 assumes that the different profiles present equal item loadings and differ in item thresholds. In addition, the within-profile IGD items co-variance is freely calculated, while the factor means are restricted to zero across the profiles. The confirmation of such an IGD model would indicate that while members of different IGD profiles use the same metric to address the items, their responses have different interpretation. Further, while there is some heterogeneity between the classes considering the IGD presentations experienced, members of different classes differ primarily regarding their item endorsement probabilities (and not their average experience of IGD behavior). Therefore, different IGD profiles have a different interpretation of the IGD dimension (in them).

FMMA-4

In the FMMA-4, item loadings, thresholds, and the item-factor co-variance are all freely estimated between the different profiles, while the factor averages are restrained to zero. The confirmation of such an IGD model would suggest that the IGD construct is addressed on a different metric and has different interpretation between the profiles, while members classified under the same profile may vary considering their IGD experience. All different FMMA variations were assessed in the present work.
