# Supplementary figures and images for: In search of the optimum structural model for Internet Gaming Disorder
Source: BMC Psychiatry. 2021 Apr 1;21:176. doi: 10.1186/s12888-021-03148-8 (PMC8015185; doi:10.1186/s12888-021-03148-8)

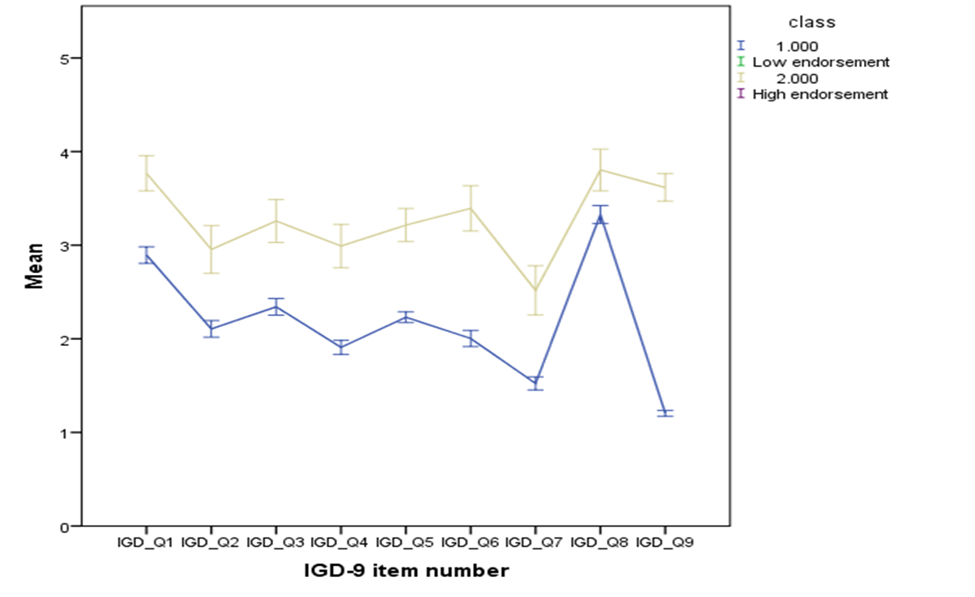

Supplement: Supplementary file 2 — Additional file 2. [file 12888_2021_3148_MOESM2_ESM.tif]
